# Oxygenation Practices During General Anesthesia in Pediatric Patients: An International Survey in Europe, USA, Australia, and New Zealand

**DOI:** 10.1111/pan.15139

**Published:** 2025-06-09

**Authors:** Jan J. van Wijk, Sanne E. Hoeks, Irwin K. M. Reiss, Robert Jan Stolker, Lonneke M. Staals

**Affiliations:** ^1^ Department of Anesthesiology Erasmus MC Sophia Children's Hospital, University Medical Center Rotterdam Rotterdam the Netherlands; ^2^ Division of Neonatology, Department of Neonatal and Pediatric Intensive Care Erasmus MC Sophia Children's Hospital, University Medical Center Rotterdam Rotterdam the Netherlands

**Keywords:** hyperoxia, oxygen, pediatric anesthesia, perioperative care, surveys and questionnaires

## Abstract

**Aims:**

At present, there is a growing body of knowledge regarding the benefits and risks associated with oxygen use in medical practice. In the perioperative period, high fractions of inspiratory oxygen are used during airway management. However, oxygen can have direct toxic effects, as well as systemic effects. In different fields of medicine, protocols exist to limit the use of oxygen, for example, in the intensive care unit and emergency department. However, in pediatric perioperative care, such protocols do not exist. We conducted an international survey among pediatric anesthesiologists to assess their daily practices regarding oxygen use during non‐cardiac surgery. The objective of this survey was to determine self‐reported perioperative oxygen use across several key areas: the default oxygen settings on anesthesia machines, the prevalence of preoxygenation, the fraction of inspiratory oxygen used intraoperatively, and considerations regarding the intraoperative administration of oxygen.

**Methods:**

An online digital survey consisting of up to 21 questions in LimeSurvey was developed and sent to 5667 members of various international pediatric anesthesia societies (ESPA, APAGBI, SPA, SPANZA).

**Results:**

A total of 828 responses were received (response rate 15%). The median reported default inspiratory oxygen (FiO_2_) value of anesthesia machines was 100% (IQR 30%–100%). Preoxygenation was used by 50% of the respondents, usually with 100% oxygen. 87% of respondents reported to titrate FiO_2_ intraoperatively, mainly based on pulse oximetry values. Median standard percentage of oxygen intraoperatively was 35% (IQR 30%–40%).

**Conclusions:**

Oxygen administration practices during pediatric anesthesia are hardly regulated. There are opportunities to further limit the use of oxygen. For instance, default settings can be lowered, and intraoperative FiO_2_ can be further titrated, mainly based on SpO_2_.

## 1 Introduction

Traditionally, due to the lack of adequate monitoring equipment, high fractions of inspiratory oxygen (FiO_2_) were used in the perioperative period to prevent hypoxemia. With the advent and use of advanced monitoring technologies, clinicians are able to monitor oxygen saturation levels more closely. Despite these monitoring technologies, the use of supplemental oxygen in pediatric care remains a subject of ongoing debate. In recent decades, more has become known about the disadvantages of oxygen [1, 2, 3, 4]. Supplemental oxygen has been identified as a risk factor for various complications, including bronchopulmonary dysplasia, necrotizing enterocolitis, intraventricular hemorrhage, and retinopathy of prematurity [5]. It has other systemic effects as well, like oxygen toxicity in the central nervous system, lungs, and cardiovascular system. This toxicity primarily arises from the formation of Reactive Oxygen Species (ROS). Additionally, ROS have been associated with potential DNA damage [6].

A shortage or surplus of oxygen in blood is referred to as hypo‐ and hyperoxemia, respectively. Although cut‐off values for hypoxemia have not been established, a PaO_2_ < 80 mmHg is generally considered abnormal. Similarly, hyperoxemia lacks a defined threshold, and there is no established safe limit for supplemental oxygen administration [7, 8, 9].

Yet progress has been made in reducing the amount of oxygen used in pediatric care. Examples include newborn resuscitation, neonatal and pediatric intensive care units (ICU), and the emergency department [10, 11, 12]. In the perioperative period, a high FiO_2_ is mostly used during the induction of anesthesia to reduce the risk of hypoxia during airway management and before extubation [13]. Especially in neonates, this requires attention since this population is both vulnerable to hypoxia and hyperoxia [14]. Furthermore, the World Health Organization (WHO) recommended in 2018 in their guidelines to use a high FiO_2_ (*f* = 0.8) to minimize the risks of postoperative surgical site infections [15]. However, the evidence for and adherence to this recommendation is limited [16, 17], and its applicability in pediatric care is actually unknown.

In 2008, a British‐Irish survey was done among members of their pediatric anesthesia association on the use of oxygen in perioperative care for neonates and infants. It demonstrated that there is still a lot of variation in the administration of supplemental oxygen [18].

A recent randomized trial compared low dose of oxygen (23% during induction and maintenance) with high dose of oxygen (80% during induction and 40% during maintenance) in 35 newborn infants with a postconceptional age below 44 weeks [19]. The authors concluded that low‐dose oxygen is safe, while high dose oxygen leads inevitably to hyperoxemia. This is consistent with a growing body of evidence in intensive care units and emergency medicine that is moving in the direction of decreasing FiO_2_ administration. However, perioperative care lacks established guidelines for oxygen titration.

To contribute to the ongoing debate, we conducted a survey among anesthesiologists who work in pediatric healthcare, in both general and pediatric centers, to gain insight on the current practice of supplemental oxygen administration during pediatric perioperative care.

## 2 Methods

The local Medical Ethics Committee (Medical Ethics Committee (MEC), Erasmus MC, Rotterdam, the Netherlands) waived the need for application of the Medical Research Involving Human Subjects Act (WMO), due to the nature of the study design and because no patients were included (April 2021, MEC number: MEC‐2021‐0212).

The primary study objective was to determine the FiO_2_ used by anesthesiologists during pediatric anesthesia, in both general and pediatric centers.

Secondary endpoints were to determine the incidence of preoxygenation, to evaluate default values of anesthesia machines, to evaluate which physiologic variables influence the administration of oxygen, and to evaluate practice patterns between (sub)groups of anesthesiologists.

An online digital prospective survey consisting of up to 21 questions was built in LimeSurvey (LimeSurvey GmbH, Hamburg, Germany). During its design stage, it was tested and evaluated by the 20 staff members of the Department of Anesthesiology of Erasmus MC Sophia Children's Hospital, Rotterdam, The Netherlands. Following review by the societies mentioned below, this survey was sent as part of the newsletter to: anesthesiologists subscribed to the newsletter(s) of the European Society for Pediatric Anaesthesiology (ESPA, 810 recipients), the Association of Pediatric Anesthetists of Great Britain and Ireland (APAGBI, 1051 recipients), the Society for Pediatric Anesthesia (SPA, 3424 recipients) and/or the Society for Pediatric Anesthesia in New Zealand and Australia (SPANZA, 382 recipients). A reminder was sent in a consecutive newsletter. No prior sample size calculation was performed since we tried to reach as many respondents as possible. For logistical and formatting reasons, the survey which was added to the SPA newsletter was presented differently, though the content remained unchanged compared to the survey in the other newsletters. The complete survey is available as supplemental content. It was online from February 9, 2022, until October 20, 2022.

All data retrieved from the participants was deidentified and put in a database. The database has been stored on the servers of the Department of Anesthesiology of Erasmus MC. All data will stay at Erasmus MC.

This manuscript adheres to the applicable CROSS guidelines.

### 2.1 Statistical Analysis

Descriptive analyses were used for describing the primary and secondary outcomes. Data were assessed for normality by visual inspection of histograms and Q–Q plots in combination with the Kolmogorov–Smirnov test. Differences between groups (e.g., general, academic, and pediatric anesthesiologist) were assessed with ANOVA or Kruskal–Wallis test. Categorical data were described with numbers and percentages, and differences were tested with Chi‐square tests. Missing data were reported, and no imputation was performed. All tests are two‐tailed, and the significance level was set at *p* < 0.05. Statistical analysis was performed with IBM SPSS Statistics (IBM SPSS Statistics version 26.0.1.0, IBM Corp, New York, NY, USA).

## 3 Results

Our survey was sent to 5667 recipients connected to the mailing lists of ESPA, APAGBI, SPA, and SPANZA. We received 828 responses, yielding a response rate of 15%. The baseline characteristics of the responders are outlined in Table 1. 78% of the responders (*n* = 648/828) had a self‐reported background in pediatric anesthesiology. Furthermore, 94% (*n* = 617/658) indicated that pediatric cases constituted over 20% of their daily anesthesia practice. 54% of the responders (*n* = 445/828) were working in a children's hospital.

**TABLE 1:** Respondents' characteristics.

	*n* (%)
**Background**	
General	258 (31.2)
Pediatric	648 (78.3)
Thoracic	42 (5.1)
Pain	34 (4.1)
Intensive care	53 (6.4)
Obstetrics	40 (4.8)
Other	49 (5.9)
**Years experience**	
< 10 years	144 (21.9)
10–20 years	266 (40.4)
> 20 years	249 (37.8)
Missing	169
**Part pediatric anesthesiology**	
< 20%	41 (6.2)
20%–80%	208 (31.6)
> 80%	409 (62.2)
Missing	170
**Type of hospital**	
Pediatric	445 (53.7)
General	383 (46.3)
**Membership**	
APAGBI	145 (17.5)
ESPA	72 (8.7)
SPA	461 (55.7)
SPANZA	21 (2.5)
National	72 (8.7)

*Note: n* = 828.

Table 2 summarizes the answers of the survey.

**TABLE 2:** Respondents' answers.

	All (*n* = 828)	Pediatric (*n* = 445)	General (*n* = 383)	*p*	Missing
Default O_2_ value anesthesia machine (%)	100% (30–100)	100% (30–100)	100% (30–100)	0.421	329 (39.7)
Use of preoxygenation (yes)	414 (50.0)	246 (55.3)	168 (43.9)	0.329	98 (11.8)
Inspiratory O_2_ (%)	100% (100–100)	100% (100–100)	100% (100–100)	0.423	3/414 (0.7)
Titration of O_2_ during preoxygenation (yes)	41 (10.0)	24 (9.8)	17 (10.4)	0.868	4/414 (1.0)
Based on saturation	14 (34.1)	8 (33.3)	6 (35.3)		
Based on expiratory O_2_	20 (48.8)	11 (45.8)	9 (52.9)	0.623	**0/41**
Based on other	7 (17.1)	5 (20.8)	2 (11.8)		
Titration of O_2_ intraoperatively (yes)	686 (87.4)	389 (87.4)	297 (87.4)	0.979	43 (5.2)
Based on saturation/pulse oximetry	522 (76.1)	325 (83.5)	197 (66.3)	**< 0.001**	**0/686**
Based on expiratory O_2_	122 (17.8)	81 (20.8)	41 (13.8)	**0.02**	**0/686**
Based on patient related factors	279 (40.7)	186 (47.8)	93 (31.3)	**< 0.001**	**0/686**
Based on other	38 (5.5)	29 (7.5)	9 (3.0)	**0.004**	**0/686**
Standard O_2_ intraoperatively (%)	35% (30–40)	35% (30–40)	40% (30–50)	0.158	109 (13.2)
Lower limit O_2_ intraoperatively (%)	25% (21–30)	25% (21–30)	30% (21–30)	0.357	119 (14.4)
Upper limit O_2_ intraoperatively (%)	50% (40–60)	50% (40–60)	50% (40–60)	0.887	143 (17.3)
PaO_2_ use for O_2_ management (yes)	523 (63.2)	335 (75.3)	188 (49.0)	**0.034**	155 (18.7)
Use of threshold SpO_2_ to reduce FiO_2_ (yes)	324 (47.3)	209 (47.0)	115 (47.9)	0.812	143 (17.3)
Which threshold (%)	96% (94–98)	96% (93–99)	96% (92–100)	0.470	9/324 (2.8)
Inspiratory O_2_ during emergence (%)	100% (80–100)	100% (80–100)	100% (80–100)	0.579	142 (17.1)

*Note:* *n* (%) or median (IQR). Bold values are Significance level *p* < 0.05.

Default value of FiO_2_ of the anesthesia machine was generally reported as 100% (IQR 30%–100%). This is the standard setting of the anesthesia machine, once it is tested and ready to use. The survey indicated that 50% of the respondents (*n* = 414/828) used preoxygenation, predominantly using a 100% oxygen concentration. Ten percent of the anesthesiologists (*n* = 41/414) titrated the FiO_2_ during preoxygenation, usually based on expiratory oxygen analysis (49%, *n* = 20/41). There were no significant differences in these results between anesthesiologists working in a children's hospital and those who are working in a general hospital (see Table 2).

Titration of oxygen intraoperatively is done by the majority of anesthesiologists (87%, *n* = 686/828). However, anesthesiologists working in a children's hospital report different and multimodal strategies more often in how to titrate supplemental oxygen. For example, they used pulse oximetry analysis and expiratory O_2_ analysis to adjust FiO_2_ more frequently compared to anesthesiologists working in a general hospital (84 vs. 66%, *p* < 0.001 and 21 vs. 14%, *p* = 0.02, respectively).

A few respondents reported in the open‐text responses to adjust FiO_2_ based on advanced monitoring like near infrared spectroscopy (NIRS) and/or transcutaneous monitoring of oxygen (tcPO_2_).

Standard FiO_2_ intraoperatively was reported to be approximately 35% (IQR 30%–40%) with a lower and upper limit of 25% (IQR 21%–30%) and 50% (IQR 40%–60%), respectively. No significant differences were observed between anesthesiologists in children's hospitals and those in general hospitals.

The survey revealed that 47% of anesthesiologists (*n* = 324/828) report reducing the FiO_2_ at a reported threshold of 96% SpO_2_ (IQR 94%–98%).

Emergence from anesthesia was performed with a FiO_2_ of 100% by all respondents, with a median FiO_2_ of 100% (IQR 80%–100%) for both children's hospitals and general hospitals (*p* = 0.56).

## 4 Discussion

This was one of the first international surveys in 15 years in the western society, offering insights into the current practice of oxygen administration in pediatric anesthesia. The use of oxygen in pediatric anesthesia still shows a notable difference compared to other medical fields, such as emergency medicine and (neonatal) intensive care medicine. In these areas, oxygen use is often more regulated and protocolled, whereas in anesthesia a broader range of approaches is still applied, indicating a less standardized practice. The reported default FiO_2_ setting for anesthesia machines is usually 100%. Moreover, when preoxygenation is used, this is done with 100% oxygen as well. Nonetheless, 87% of respondents indicate that they titrate the intraoperative FiO_2_, with the median value being around 35%. Although there are no formal (international) guidelines, the outcomes of our survey suggest there could be different possibilities for improvement in the use of supplemental oxygen in pediatric perioperative care.

Growing evidence supports the need for a revised approach to oxygen management in pediatric anesthesia. Evidence of oxygen's negative effects in various contexts suggests that a more restrictive approach to oxygen use may be preferable to a liberal policy. For instance, during neonatal resuscitation, oxygen supplementation is initially limited to 21% [20]. If clinically necessary, this is gradually raised based on the judgment of the attending pediatrician. Similarly, during advanced life support in trauma care and during pediatric resuscitation, the use of oxygen is also limited and targeted on SpO_2_ values of 94%–98% [10, 11]. Ventilation strategies in neonatal intensive care units aim to be as minimally invasive as possible, with the lowest possible amount of supplemental oxygen. Oxygen titration is done meticulously based on clinical indicators, SpO_2_ values, and arterial blood gas analysis [12, 21]. Data from a recent meta‐analysis at pediatric intensive care units demonstrated an association of hyperoxia with mortality. Therefore, the authors of this meta‐analysis suggest a more cautious approach for the use of oxygen [3].

Over time, perioperative safety has improved by developments in monitoring, equipment, and medication. However, the approach to oxygen administration may not have kept pace with these improvements. The use of nitrous oxide has declined, which means that the risk of hypoxia has been reduced. Nevertheless, anesthesiologists are continuing to use high levels of oxygen, first and foremost during the induction and emergence phase. Hypoxia during induction and emergence remains a substantial concern. Careful monitoring, as well as appropriate oxygenation strategies, can mitigate the risks of severe complications like bradycardia or cardiac arrest, especially in the context of airway management. There is very limited evidence for the feasibility of low FiO_2_ during induction and maintenance in the neonatal population, as shown in a small RCT [19]. However, the benefits of supplemental oxygenation for airway management have extensively been highlighted in recent literature, especially in neonates and infants [22, 23, 24]. A recent joint guideline on airway management in neonates and infants emphasizes this importance, with a strong recommendation on supplemental oxygen during tracheal intubation through apneic oxygenation [25]. Post‐operative nausea and vomiting (PONV) could be a reason to apply a higher FiO_2_ during anesthesia. Although extensively investigated in adults, this finding remains controversial [26]. In pediatric anesthesia, the available evidence is also pointing towards no positive effects of supplemental oxygen on PONV as well [27]. Other evidence from perioperative care shows that lower oxygen concentrations are associated with less atelectasis formation, although not every study conducted on this topic confirms this observation [28, 29, 30]. Postoperative lung function is also impaired in older children who received a higher FiO_2_ perioperatively [31].

Our results indicated differences in the intraoperative approach of oxygen titration between anesthesiologists employed in children's hospitals compared to those working in general hospitals. The survey was not designed to further investigate this result, so we could only speculate on the reasons behind it. One plausible explanation could be that anesthesiologists in children's hospitals might be more familiar with the discussion concerning the side effects of oxygen in neonatal ICUs. Another possibility might be that those who are working in children's hospitals are more at ease to work with children and, therefore, less hesitant to reduce the amount of oxygen. However, the generalizability of the results may well be possible, especially since there are no protocols to guide clinicians in the titration of supplemental oxygen in the perioperative period.

Thus far, it seems that little has changed in our approach to oxygen in pediatric perioperative care, especially when compared to adjustments in intensive care medicine and resuscitation care over recent decades. Even following the findings of a British‐Irish survey conducted in 2008, which highlighted similar concerns, the perioperative management of oxygen in pediatric patients has largely remained unchanged [18]. However, with some minor modifications, we can take small steps towards improvement. For example, it should be a small technical step to reduce the default inspiratory oxygen setting on anesthesia machines to a lower level. Apart from the physiological changes it should lead to, it could create awareness among anesthesia healthcare professionals. A lower FiO_2_ has beneficial effects on atelectasis formation, and it could, in the end, lower the risk of several aforementioned disorders (bronchopulmonary dysplasia, necrotizing enterocolitis, intraventricular hemorrhage, and retinopathy of prematurity) and the formation of ROS. Apart from changing default settings, local protocols could be made for the titration of supplemental oxygen. On the basis of the type of patients and/or type of surgery, limits could be determined when to raise or lower the FiO_2_. To our knowledge, no (inter)national protocols exist to guide the use of supplemental oxygen in pediatric perioperative care.

An important limitation of this survey was its response rate of 15%. This was expected, since physicians are known for completing surveys poorly [32]. Consequently, double responses in the data set are unlikely. A follow‐up survey gathering reasons for nonresponse was not possible due to the nature of the distribution of the initial survey. Although there were some differences in the survey sent to SPA members compared to the other societies, this had no substantive consequences, nor were there clinical differences in their response compared to the other societies. Furthermore, just over 50% of the respondents were working in a children's hospital. These clinicians may have been more likely to complete the survey due to increased concerns about oxygen management in pediatric patients. This could have influenced the results, as risk groups (i.e., neonates) are usually cared for at specialized hospitals. The representation of our results could be questioned. We have tried to reach as many respondents as possible. Our sample consists of colleagues who are connected to pediatric anesthesia and who might be familiar with this topic. Therefore, results from the entire study population could likely be more heterogeneous. However, this does not alter our conclusions; rather, it underscores the need for further action in refining oxygen use practices in pediatric anesthesia.

Further research is necessary to determine the amount of supplemental oxygen in specific types of patients and, ideally, should concentrate on assessing the outcomes associated with hyperoxemia. This includes investigating clinically relevant pulmonary complications, postoperative wound infections, length of hospital stay, and, though challenging, mortality. Thereafter, it may be necessary to develop protocols to further limit the use of supplemental oxygen. Furthermore, (inter)national pediatric anesthesia societies could come up with a consensus statement regarding the optimal use of oxygen.

## 5 Conclusions

Oxygen use during pediatric anesthesia is hardly regulated and can be improved. There are opportunities to further limit the use of oxygen. For instance, default settings can be lowered, and intraoperative FiO_2_ can be further titrated based on clinical parameters like, among others, SpO_2_. The findings of this survey, alongside other studies, may be a wakeup call to further expand and enhance this ongoing debate.

## Ethics Statement

The local Medical Ethics Committee (Medical Ethics Committee (MEC), Erasmus MC, Rotterdam, The Netherlands) waived the need for application of the Medical Research Involving Human subjects Act (WMO) because of the nature of the study design and because no patients were included.

## Consent

The authors have nothing to report.

## Conflicts of Interest

S.E. Hoeks is a statistics editor of the Editorial Board of Pediatric Anesthesia.

## Supporting information


Data S1.

## Data Availability

The study data are not publicly available due to institutional policy. Data are available upon reasonable request from the corresponding author.
