# Supplementary material for: Oxygenation Practices During General Anesthesia in Pediatric Patients: An International Survey in Europe, USA, Australia, and New Zealand
Source: Paediatr Anaesth. 2025 Jun 9;35(8):643–8. doi: 10.1111/pan.15139 (PMC12233042; doi:10.1111/pan.15139)
Supplement: Supplementary file 1 — Data S1. [file PAN-35-643-s001.zip › Survey ESPA_APAGBI_SPANZA.docx]

Introduction of questionnaire:

With this questionnaire we would like to investigate common practice according to oxygen administration during non-cardiac pediatric anesthesia among anesthesiologists in the western society. The questions are related to your daily practice when anesthetizing children (0-18 years of age) for non-cardiac surgery. Keep in mind your daily routine habits: what is your standard procedure for low-risk surgery in healthy children (ASA I-II).

Our local Medical Ethics Committee has waived the need for application of the Medical Research Involving Human subjects Act (WMO), because of the nature of the study design. Your answers will be anonymous.

Thank you in advance.

Questions:

What is the default value of inspired oxygen (percentage) of the anesthesia machines in your hospital?

(if unknown, please answer 0%)

%

Do you use preoxygenation as standard of care?

Yes / no

If yes: what percentage of inspiratory O_2_ do you generally use for prexoygenation?

%

If yes: do you titrate inspiratory O_2_ (FiO_2_) by effect during preoxygenation?

Yes / No

If yes: on what effect?

Options: (saturation / expiratory O_2_ / patient related factors such as [open box] / other [open box] )

Do you titrate FiO_2_ by effect intraoperatively?

Yes / no

If yes: on what effect?

Options: (saturation / expiratory O_2_ / patient related factors such as [open box] / other [open box] )

What is on average your standard percentage of O_2_ intraoperatively?

%

Which lower limit of inspiratory O_2_ (percentage) do you use intraoperatively (so after induction but before end of operation)?

%

Which upper limit of inspiratory O_2_ (percentage) do you use…

(Note: on average; during incidents we all will use 100% sometimes; the question is about your routine use of oxygen.)

-intraoperatively (so after induction but before end of operation)?

%

-at the end of the operation?

%

-in the recovery room?

%

Do you use a threshold in saturation (SpO_2_) to reduce FiO_2_?

Yes / no

If yes: which threshold (SpO_2_) do you use to lower FiO_2_?

[open box]

When you use arterial blood gas analysis including PaO_2_, does that influence your inspiratory O_2_ settings?

Yes / no

If yes: how does PaO_2_ influence your policy?

[open box]

General questions:

What is your background as anesthesiologist?

general / pediatric / thoracic / pain / intensive care / obstetric / other: [open box]

How many years of experience do you have as an anesthesiologist?

[numeric]

What is the percentage of pediatric anesthesiology in your daily practice?

Categorize: 0-10-20- … -100%

At what kind of hospital do you work?

general / university hospital / children’s hospital / university (affiliated) children’s hospital / private / other

In which country do you work?

[open box]

Are you a member of one of the pediatric anesthesiology associations and, if so, which one?

APAGBI / ESPA / SPA / SPANZA / National pediatric anesthesia society

Do you have any comments?

[open box]

Thank you for your time!
